# Understanding Online Registered Nursing Students’ Attitudes Towards Environmentally Sustainable Healthcare

**DOI:** 10.3390/nursrep15090340

**Published:** 2025-09-18

**Authors:** Rebecca Rawson, Uchechukwu V. Okere, Alan Williams, Geraldine Lyte, Jessica E. Jackson

**Affiliations:** 1 School of Science, University of Derby, Derby DE22 1GB, UK; u.okere@derby.ac.uk; 2 School of Nursing and Midwifery, University of Derby, Derby DE22 1GB, UK; a.williams@derby.ac.uk (A.W.); g.lyte@derby.ac.uk (G.L.); 3 School of Health Sciences, University of Nottingham, Nottingham NG7 2UH, UK; jessica.jackson1@nottingham.ac.uk

**Keywords:** environmental sustainability, nursing education, sustainable healthcare, nursing students, nursing

## Abstract

**Background/Objectives:** The healthcare sector is a significant source of pollution, and it is widely acknowledged that changes are required to transition to more sustainable healthcare practice. Nurses represent more than half of the sector’s workforce and are uniquely positioned to enact change. However, expertise on environmental sustainability within the nursing field is a barrier despite the topic being positively embraced by students. **Methods:** This research employed a cross-sectional design using an anonymous online survey with convenience sampling from registered nursing students studying online to understand their attitudes towards environmentally sustainable healthcare. Data were collected between April 2023 and January 2024 with quantitative results analysed using descriptive statistics and qualitative results using thematic analysis. **Results:** Results show that registered nursing students are aware of the negative environmental impact of healthcare practice, realise the importance of working more sustainably and understand the value and role of education to facilitate meaningful change in the sector. However, they called for more educational content, specifically on carbon footprints, waste management, and resource use, paired with organisational leadership support and workplace training in healthcare settings. **Conclusions:** Adopting these recommendations endorsed by student nurses in practice could support nurses to reduce the environmental burden of the healthcare sector and contribute to both net zero and the United Nations Sustainable Development Goals.

## 1. Introduction

The healthcare sector, which aims to protect and promote health, is a significant source of pollution. It is energy and resource intensive and contributes to global waste generation and greenhouse gas emissions. Health Care Without Harm [1] reports that the collective impact of the global healthcare sector would rank it as the fifth largest emitter if it were a country. It is now widely acknowledged that a change in approach to healthcare practice is required to transition to more sustainable development [2,3,4,5,6]. Sustainable development as defined by the 1987 Brundtland Report is “Development that meets the needs of the present without compromising the ability of future generations to meet their own needs.” [7]. This has been refined by the World Health Organization [8] as an environmentally sustainable “health system that improves, maintains or restores health, while minimising negative impacts on the environment and leveraging opportunities to restore and improve it, to the benefit of the health and well-being of current and future generations”. Kaplan and Forst [9] further specify that this should encompass “leaner energy, less waste, safer chemicals, smarter purchasing, healthier food, and engaged leadership”.

Nursing staff represent more than half of the healthcare workforce [10] and collectively they are in a key position to both influence and enact change [11,12,13]. Shaban et al. [14] (p. 13) state that when nurses foster more sustainable practices, they “contribute to better health outcomes, responsible resource management, and climate change mitigation.” Further, Rosa et al. [15] have pointed out that for the United Nations (UN) Sustainable Development Goals (SDGs) to be realised, nurses and midwives will need to leverage their roles and responsibility on a global scale as advocates, leaders, clinicians, scholars, and full partners across health systems. In response to these calls for action the International Council of Nurses published a position statement in 2024 [16] acknowledging the importance of reducing the impact of healthcare and calling on nurses to support more environmentally sustainable practice in the sector. In the United Kingdom (UK), the Nursing and Midwifery Council [17] have also started to actively address sustainability within the healthcare sector, and in April 2024, they launched their inaugural Environmental Sustainability Plan, demonstrating a commitment to reducing their own environmental impact and promoting sustainable practices among nurses, midwives, and nursing associates. This plan also aims to build resilience against climate-related risks within the organisation. However, Walpole et al. [18] have called for all health and social care professional standards to be updated to advance leadership and action for environmental sustainability and planetary health.

Despite these commitments, nurses have identified barriers in their attempts to engage in adequate environmental behaviors in their workplace, including time, resources, support, and staffing [19]. It is therefore paramount that nursing education prepares the workforce to both manage and mitigate impacts to foster environmental conscious practice. As such, education, which reinforces the importance of reducing the sector’s environmental impact whilst supporting the achievement of net zero goals and UN SDGs, is needed. Indeed, Shaban et al. [14] found that “ecoconscious nursing”, which encompasses reducing waste, managing resources, and conserving energy, will support the achievement of five of the 17 UN SDGs; SDG 3 (Good Health and Well-being), SDG 6 (Clean Water and Sanitation), SDG 7 (Affordable and Clean Energy), SDG 12 (Responsible Consumption and Production), and SDG 13 (Climate Action).

In nursing education, Siemon et al. [20] have acknowledged that current content is shifting to include environmental and sustainability issues, with Richards et al. [21] suggesting that climate change is an essential component. This is reinforced by Shaban et al. [14] who consider environmental sustainability to be a core competency for nursing education. Barna et al. [22], however, caution that there is a paucity of nursing educators with the required expertise to achieve this, presenting a significant barrier to the effective integration of key environmental sustainability issues within nursing education.

Several research studies have explored the views, attitudes, and opinions of nursing students regarding the changing climate and their education. Álvarez-Nieto et al. [23], for example, investigated undergraduate nursing students’ attitudes on sustainability and their views on its inclusion in the curriculum and reported a positive outlook on this from their student sample. Previous studies, however, have largely focused on campus-based students [24,25,26] and have not explored how registered nurses of all fields who are already working in practice would like to see environmental sustainability integrated into their training and area of practice. This research therefore draws on the views of registered practice nurses studying online for either an undergraduate or postgraduate degree at a UK University. The current inclusion of environmental sustainability content in the course materials for these students is limited.

The aim of this study was therefore to understand the students’ attitudes towards environmentally sustainable healthcare, and determine what environmental sustainability practices they have experienced in the workplace and what environmental sustainability content they would find valuable in their university studies.

## 2. Materials and Methods

### 2.1. Sample and Setting

This cross-sectional study was conducted at the University of Derby (UK) using an anonymous online survey. The target population comprised all registered nursing students enrolled on the online nursing programs. These students are qualified international nurses who are practicing in their country of residence. Convenience sampling was used. After data cleaning (removal of missing values), 59 student responses were retained for analysis.

### 2.2. Data Collection

Students were informed about the study via announcements on their program pages within the university’s virtual learning environment and by emails sent to their student accounts. Recruitment materials contained a link to the Qualtrics^®^ survey, which included a downloadable Participant Information Sheet. Participants provided informed consent electronically before starting the survey. The survey was open for nine months (April 2023–January 2024). No compensation was offered.

### 2.3. Instrument

The survey consisted of 14 items.

- Items 1–4 captured student characteristics (e.g., time in practice, workplace role).
- Items 5 and 7–13 were closed-ended Likert-scale questions assessing views on sustainability priorities in practice and education, as well as experiences with related initiatives. Each Likert item was followed by an open-ended question inviting students to explain their reasoning.
- Item 6 asked students to select their top five priority areas for nursing from the 17 UN Sustainable Development Goals (SDGs).
- Item 14 asked which aspects of sustainability students would like to learn more about.

The full instrument is provided in Appendix A.

### 2.4. Data Analysis

The online survey was hosted in Qualtrics^®^ and the results were downloaded to a Microsoft Excel^®^ spreadsheet. Following data cleaning (the removal of missing values), a total of 59 students’ responses were analysed. Descriptive statistics were used to interpret the quantitative data (closed questions), with the results presented in graphs and charts. Qualitative results (open questions) that included an expression of opinion were interpreted using thematic analysis. Braun and Clarke’s [27] six-step guidelines were followed, and the process is detailed below:

1. Familiarisation with the data—the qualitative responses were added to a Microsoft Word^®^ document where they were read multiple times to allow immersion in the data to identify patterns.
2. Initial codes generation—codes in the form of words and short phrases were manually assigned to each of the responses using the comment facility on Microsoft Word^®^. An inductive approach was used with no pre-determined codes, and sixteen codes were identified across the data set.
3. Search for themes—the coded data were reviewed, and the Microsoft Word^®^ highlighter tool was used to identify commonalities and patterns across the data set. Each code was then organised into a group and attached to a theme using a mind map process.
4. Review themes—the themes identified from collating the codes were reviewed and refined.
5. Naming themes—the themes were named so that they represented a clear account of the data they presented
6. Writing the report.

Some of the qualitative survey questions did not, however, obtain enough written data to include the results in the thematic analysis. These responses were therefore tallied to identify commonalities, for example, where students shared examples using key words or phrases, these were collated into groups and are either discussed in the main text or were used to produce graphs and charts.

#### Ethical Considerations

Ethical approval was granted by the University of Derby Research Ethics Committee (ETH2122-2239). Participation was voluntary with electronic informed consent, and the survey was anonymous. No incentives were provided.

## 3. Results

### 3.1. Student Participant Characteristics

Of the 59 survey responses included for analysis, 75% were from UK-based registered nursing students and 25% were from international registered nursing students. A total of 80% of the registered nurses were working in in-patient care, with the remaining 20% in outpatient care. There was a broad spread for the number of years of workplace experience within the sample, ranging from 0 to 21+ years, with the largest proportion, 29%, having worked as a registered nurse for six to ten years (see Table 1).

### 3.2. Quantitative Findings

#### 3.2.1. Student Views on Sustainability in Healthcare and Education

**Figure 1:**
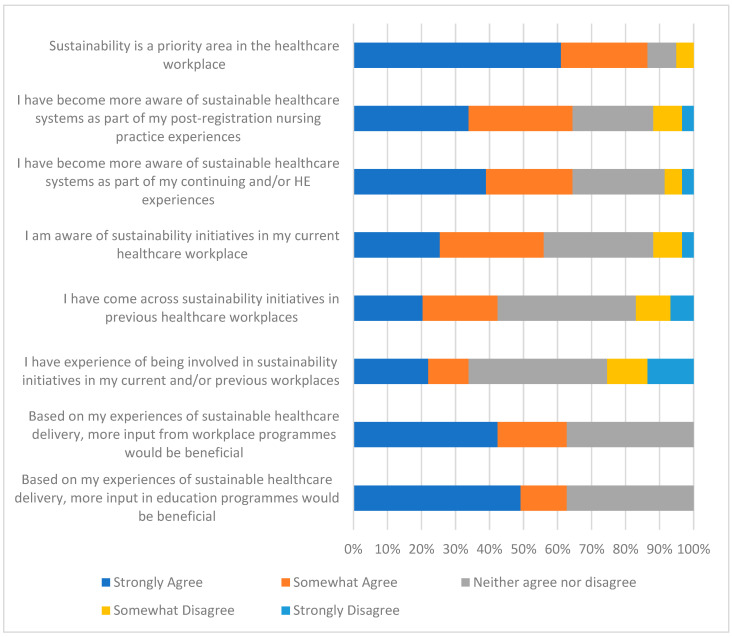
Student views regarding sustainability in nursing practice and education.

Figure 1 presents students’ perspectives on the role of sustainability in healthcare and education. The majority (86%, n = 52) agreed that sustainability is a priority in healthcare. Over half reported greater awareness of sustainable healthcare systems through post-registration practice (64%, n = 38) and through higher education (64%, n = 38). Awareness of workplace initiatives was higher for current workplaces (56%, n = 33) than for previous workplaces (42%, n = 25). Only 34% (n = 20) reported personal involvement in sustainability initiatives. Nevertheless, 63% (n = 37) believed sustainability initiatives would be beneficial in their workplace. Similarly, 63% (n = 37) agreed that more education on sustainability in healthcare should be integrated into nursing programmes.

#### 3.2.2. Examples of Workplace Sustainability Initiatives

**Figure 2:**
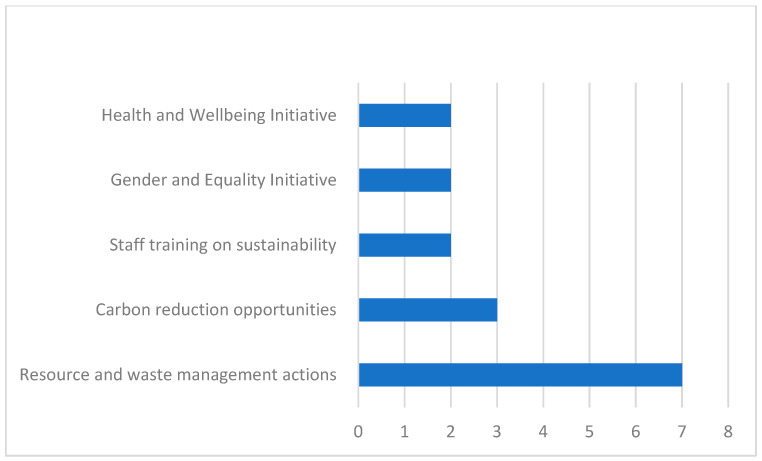
Examples of current workplace sustainability initiatives.

Among the 56% (n = 33) of students aware of initiatives in their current workplace, 16 provided examples (Figure 2). The majority related to resource and waste management (n = 7), such as water-saving mechanisms, waste elimination, and recycling promotion. Other examples included education and awareness activities. For previous workplaces (n = 25), 11 students shared examples: most referred to training initiatives (n = 6), followed by resource/waste management (n = 3) and gender and equality initiatives (n = 2).

#### 3.2.3. Views on Education for Sustainability

Of the 37 students (63%) who supported more education on sustainability in healthcare, 27 elaborated on expected benefits: improved staff competence (n = 18), improved healthcare quality (n = 7), improved efficiency (n = 1), reduced healthcare costs (n = 1).

#### 3.2.4. Areas of Sustainability Students Wish to Learn More About

Of the 35 students who answered this question, 20% (n = 7) highlighted environmental sustainability topics such as reducing waste (n = 3), responsible consumption (n = 1), carbon footprints (n = 1), and energy use (n = 2). The remaining students expressed interest in economic sustainability, healthcare system longevity, and staff/patient wellbeing.

#### 3.2.5. UN SDG Priority Areas for Nursing Students

**Figure 3:**
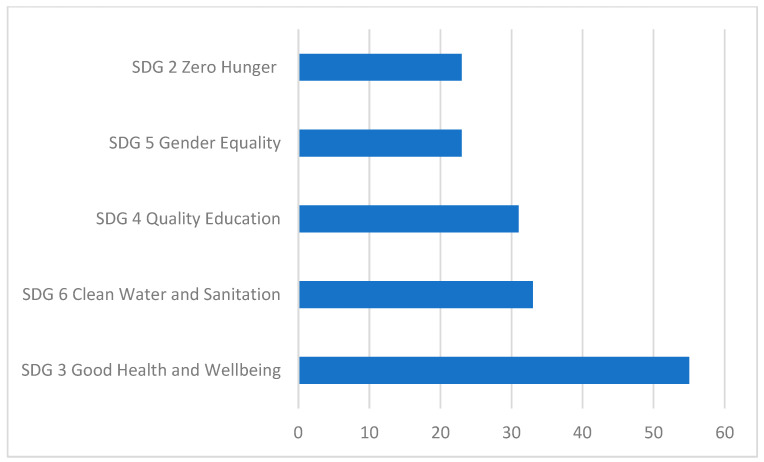
Top five priority areas for nursing from the UN SDGs (overall tally).

Students chose five out of the seventeen UN SDGs that they felt were priority areas for nursing (see Figure 3). SDG3 Good Health and Wellbeing was reported at a significantly higher rate (93%, n = 55) than all other goals. The remaining top four priority areas identified by nursing students were SDG 2 Zero Hunger (38%, n = 22); SDG 4 Quality Education (53%, n = 31); SDG 5 Gender Equality (39%, n = 23), and SDG 6 Clean Water and Sanitation (56%, n = 33).

### 3.3. Qualitative Findings

**Figure 4:**
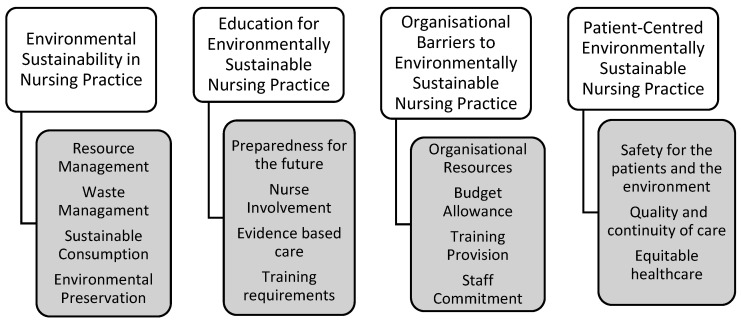
Final themes and their associated codes.

The results from five of the open-ended survey questions were coded and grouped into four overarching themes. These were (1) Environmental Sustainability in Nursing Practice; (2) Education for Environmentally Sustainable Nursing Practice; (3) Organisational Barriers to Environmentally Sustainable Nursing Practice; and (4) Patient-Centred Environmentally Sustainable Nursing Practice. Figure 4 illustrates the four themes identified in this data set alongside the codes that informed them.

#### 3.3.1. Environmental Sustainability in Nursing Practice

It was evident from the responses that sustainability in healthcare practice was seen as a priority area with specific emphasis placed on waste reduction, resource use, and preserving the environment. Indeed, students reported that “it is paramount [to] avoid wastage”; that they should “ensure resources are maximised and utilised reasonably” and it will “help to improve efficiency”. Recycling and reusing were frequently highlighted alongside the importance of minimising the use of single-use equipment. There were, however, some positive observations of these elements already in practice. Students stated that they had either observed or been involved in resource-saving measures when “sterility was not required”; “recycling projects”, and “segregation of waste initiative[s]”. Students also acknowledged the role of nurses as change agents, stressing that “nurses carry out their responsibilities with an aim of achieving the sustainable development goals” and that “nurses can play a significant role in promoting sustainable healthcare systems by incorporating environmentally friendly practices into their daily routines”. However, it was also stressed that sustainability was “everyone’s responsibility, not just nursing, or healthcare” and that community education and awareness would be vital to achieving sustainable outcomes.

#### 3.3.2. Education for Environmentally Sustainable Nursing Practice

There was a clear consensus among students that education and training were essential for more sustainable nursing practice, not only to reduce the impact of healthcare on the environment but to also reduce costs and minimise the impact of climate change on human health. Indeed, with reference to the workplace, students stated, “it is important that sustainability be prioritised to reduce the cost of running health facilities and reduce the impact of healthcare waste on the environment” and “nurses should be involved in global health matters such as climate change […] raising awareness of its negative impact on health”. Further, “education to trainees”; “divisional days”; “workplace programs” and “retraining of staff” were all suggestions for addressing education gaps in the workplace.

Regarding nursing education in HE, a comparable attitude was apparent with students noting that “teaching sustainability should be embedded into everything we do”; and that “education empowers” and “is key to success”. There was, however, acknowledgement that sustainability in the current learning material at the university was deficient. One student for example, stated that “it is not made a priority” whilst others reported that there is “not much included in the modules” and “only some brief information [is] given”. This was further supported by one student who noted “there is a need for sustainability in education in health care to catch up with current evidence-based care”.

#### 3.3.3. Organisational Barriers to Environmentally Sustainable Nursing Practice

Students stressed that there were barriers preventing full engagement with sustainable healthcare practice in the workplace, including cost, time, resources, management support, and both staff knowledge and inclination to engage. Students for example, stated that it is “not really promoted well” and “not currently visible” whilst others felt there was a “lack of funding” or “funding […] was not encouraging” and that there was “much that could be done to reduce waste”. Indeed, one response stated, “recycling in my department is STILL not possible” and another reported any sustainable initiatives “are always short term”. The importance of a whole organisation approach was reported with students noting that it “needs the input and buy-in from other departments”; would require “continuous support”, and that “managers and leaders should support” to ensure it is successful. Students also expressed personal disappointment in their attempts to engage in more sustainable nursing practice at work. One said, “it is sometimes difficult to persuade others to follow your example” whilst another said, “[I] feel alone in my attempt”. This was further compounded by a third student who voiced “when I have spoken up in my workplace about ideas or new things I have come across. I always get shot down and I am made to feel out casted”.

#### 3.3.4. Patient-Centred Environmentally Sustainable Nursing Practice

The importance of aligning sustainability with quality healthcare practice to ensure patient safety and an equitable service was echoed by students. Delivering “continuity of care” and “top value care” was expressed alongside views that “nurses should perform procedures in a safe manner, so as to protect and preserve the environment”. Further one student declared that “environmental quality is as important as the care given to patients”. The relationship between sustainable practice and positive healthcare outcomes was acknowledged by several students as well as the importance of ensuring growing populations and future generations had access to “safe and efficient care”.

## 4. Discussion

The aim of this study was to understand registered nursing students’ attitudes towards environmentally sustainable healthcare, and to determine what environmental sustainability practices they have experienced in the workplace and what environmental sustainability content they would find valuable in their online HE studies. This study’s findings show that registered nursing students within online HE are aware of the negative impact of healthcare practice on the environment, realise the importance of working more sustainably to reduce this impact, and understand the value and role of education to facilitate meaningful change in the sector.

The environmental sustainability practices experienced by students in either their current or previous workplace largely consisted of waste and resource management strategies, and whilst this presents a positive insight into initiatives, as less than half of students were aware of any actions in their workplace, there is significant scope for improvement. Further, when considered alongside the barriers to sustainable practices highlighted, including an absence of top-down support, funding obstacles, and poor staff engagement, it is evident that stronger organisational commitment is needed. As such, students called for more inclusion of sustainability priorities in the workplace, with a specific request for more direction and support from management. This corroborates the findings of Saleem et al. [28] who emphasised the importance of sustainability driven leadership and management-led actions to inspire staff to work more sustainably. Students also raised the importance of workplace training for all staff to foster a more sustainable outlook and increase the uptake of engagement in sustainable practices across the sector. These suggestions align with previously published recommendations for actions for sustainability in healthcare [29,30,31].

Students emphasised the importance of environmental sustainability content in nursing education, which concurs with both Richards et al. [21] and Shaban et al. [14]. However, as warned by Barna et al. [22], this study’s findings show that the current HE learning experiences of this group of online students did not fully embed sustainability, indicating an area for development in the current nursing curricula. That said, in line with Álvarez-Nieto et al. [23], students were keen to see more environmental sustainability content in their studies. The specific requests for education on waste management, resource use, and carbon footprints, reaffirm views on the importance of sustainable healthcare practice and present a set of recommendations for course content inclusion in all HE nursing programs. It also aligns with the principles of ecoconscious nursing [14]. It should however be noted that this line of questioning in the survey fostered different interpretations of sustainability. Indeed, there were several requests for learning content on the durability of the healthcare sector from a financial and staff and patient wellbeing perspective. This offers an insight into the broader sustainability perceptions of healthcare staff and indicates the focus on patient care and future proofing in healthcare settings.

Finally, the results show that students are aware of their collective influence as change agents for more sustainable healthcare practice, cementing their position as a force to both influence and deliver [8,9]. This is endorsed by the Centre for Sustainable Healthcare [32], who emphasise the “crucial role” of nurses in reducing healthcare impacts and support the achievement of net zero goals. The SDGs, especially Goal 3, Good Health and Well-being, aim to ensure healthy lives and promote well-being for all. It is therefore encouraging that the students identified Goal 3 as the highest priority area for the sector. Progress in this SDG will reflect improvements in healthcare access, quality, and outcomes globally. Patient care is central to achieving these health-related goals because quality care directly impacts patient outcomes, reduces mortality, prevents disease, and improves quality of life. Effective patient care ensures that health systems are responsive, equitable, and able to meet the needs of diverse populations, which is crucial for advancing SDG targets like reducing maternal and child mortality, combating infectious diseases, and promoting mental health.

The outcomes of this study are valuable as they reflect the environmental sustainability experiences, perceptions, and recommendations of registered nurses studying online, whereas previous research has focused on campus-based programs and pre-registered nurses. Online learning is a valuable tool for the nursing profession [33], providing flexible HE to a global body of learners, as indicated by both the national and international students in this study’s sample. As such, integrating the recommended environmental sustainability content into online courses will prepare nursing students to reduce the environmental impact of the sector alongside their essential role in patient care and support them to address their identified barriers to more sustainable healthcare practice.

*Strengths and Limitations*

This study contributes to the emerging evidence base on sustainability within nursing education and practice by capturing perspectives from both UK-based and international registered nursing students. The cross-sectional design and use of an online survey facilitated wide accessibility, while combining closed- and open-ended questions enabled collection of both measurable data and personal insights. Ethical safeguards and a transparent analytic process further strengthen the study’s rigour.

However, several limitations must be acknowledged. The reliance on convenience sampling from a single UK university limits the generalisability of findings. Participants may not represent the broader population of registered nurses across different institutional and cultural contexts. Only 59 responses were obtained over a nine-month period, which may limit representativeness. While the survey included fourteen questions, the balance between closed and open-ended items may not have fully captured the complexity of students’ attitudes and experiences. Qualitative analysis was conducted manually in Microsoft Word^®^. Although systematic, the absence of dedicated qualitative data analysis software (e.g., NVivo, Atlas.ti) may have reduced coding consistency and limited the audit trail. These limitations suggest caution when interpreting the findings; nevertheless, the study provides valuable preliminary insights that can inform future, larger-scale research. Further, the survey did not explicitly define environmental sustainability, and as such, the survey focus could be misinterpreted. Future research should therefore seek to engage a wider range of students utilising different sampling strategies and focus on a specific environmental sustainability line of questioning.

## 5. Conclusions

This study examined the perspectives of registered nursing students enrolled in online HE programs regarding environmental sustainability in healthcare. The findings indicated that more than half of the students reported awareness of sustainability initiatives in their workplaces; most focused on waste and resource management, while others cited education and equality-related programs. Many students expressed a desire for more education on sustainability in healthcare, anticipating benefits such as improved competence, quality of care, and efficiency. Students also identified priority areas for further learning, including reducing waste, carbon footprints, and energy use and selected UN SDGs most relevant to nursing, with SDG 3 (Good Health and Wellbeing) highlighted by 93% of participants. Qualitative themes included: (1) Environmental Sustainability in Nursing Practice; (2) Education for Environmentally Sustainable Nursing Practice; (3) Organisational Barriers to Environmentally Sustainable Nursing Practice; and (4) Patient-centred Environmentally Sustainable Nursing Practice.

These findings underscore both the growing awareness among nursing students of the environmental challenges facing healthcare and the barriers they face in translating this awareness into practice. Based on these insights, the study recommends that HE programs integrate sustainability topics, such as waste reduction, resource efficiency, and carbon literacy, into curricula. Healthcare organisations should provide leadership support and workplace training to reinforce sustainable practice and remove structural barriers. Together, these measures can enhance nurses’ capacity to contribute to environmentally sustainable healthcare and support progress towards the UN SDGs and net-zero targets.

## Figures and Tables

**Table 1:** Student participant characteristics.

Characteristic	n	%
**Location**		
UK-based nursing students	44	75
International nursing students	15	25
**Workplace Setting**		
In-patient care	47	80
Out-patient care	12	20
**Years of Experience as Registered Nurse**		
0–5 years	9	15
6–10 years	17	29
11–15 years	12	20
16–20 years	10	17
21+ years	11	19

## Data Availability

The data presented in this study are available on request from the corresponding author.

## Public Involvement Statement

No public involvement in any aspect of this research.

## Guidelines and Standards Statement

This manuscript does not follow a specific EQUATOR Network reporting guideline because the study design does not align with the major categories covered (e.g., randomised trials, observational epidemiology, qualitative interviews, systematic reviews). The project involved student participants reflecting on and reporting their own experiences of nursing practice, which is more akin to an educational evaluation or descriptive survey rather than a clinical or biomedical research design. For transparency and clarity, we have nevertheless reported methods, participant characteristics, data collection, and analysis procedures in detail, consistent with general principles of rigorous reporting.

## Abbreviations

The following abbreviations are used in this manuscript:

HE	Higher Education
SDG	Sustainable Development Goal
UK	United Kingdom
UN	United Nations

## Appendix A

### Survey Questions

1. How many years of workplace experience do you have as a registered nurse?
  0–5; 6–10; 11–15; 16–20; 21+
2. What country do you practice in?
3. What is your current area of practice (for example hospital: medicine, surgery, intensive care; community; private or voluntary; education; research; management)?
4. What is your current job role?
5. Sustainability is a priority area in the healthcare workplace.
  Strongly Agree; Agree; Neutral; Disagree; Strongly Disagree
  Please add some further detail on your views here
6. The United Nations has 17 Sustainable Development Goals outlining priority areas for global action for sustainability. Please choose the top five priority areas for nursing from the list below:
  - Goal 1 End poverty in all its forms everywhere
  - Goal 2 End hunger, achieve food security and improved nutrition and promote sustainable agriculture
  - Goal 3 Ensure healthy lives and promote well-being for all at all ages
  - Goal 4 Ensure inclusive and equitable quality education and promote lifelong learning opportunities for all
  - Goal 5 Achieve gender equality and empower all women and girls
  - Goal 6 Ensure availability and sustainable management of water and sanitation for all
  - Goal 7 Ensure access to affordable, reliable, sustainable and modern energy for all
  - Goal 8 Promote sustained, inclusive and sustainable economic growth, full and productive employment and decent work for all
  - Goal 9 Build resilient infrastructure, promote inclusive and sustainable industrialisation and foster innovation
  - Goal 10 Reduce inequality within and among countries
  - Goal 11 Make cities and human settlements inclusive, safe, resilient and sustainable
  - Goal 12 Ensure sustainable consumption and production patterns
  - Goal 13 Take urgent action to combat climate change and its impacts
  - Goal 14 Conserve and sustainably use the oceans, seas and marine resources for sustainable development
  - Goal 15 Protect, restore and promote sustainable use of terrestrial ecosystems, sustainably manage forests, combat desertification, and halt and reverse land degradation and halt biodiversity loss
  - Goal 16 Promote peaceful and inclusive societies for sustainable development, provide access to justice for all and build effective, accountable and inclusive institutions at all levels
  - Goal 17 Strengthen the means of implementation and revitalise the global partnership for sustainable development
7. I have become more aware of sustainable healthcare systems as part of my post-registration nursing practice experiences
  Strongly Agree; Agree; Neutral; Disagree; Strongly Disagree
  If you agree with the above statement, please give an example from your experience
8. I have become more aware of sustainable healthcare systems as part of my continuing and/or higher education experiences
  Strongly Agree; Agree; Neutral; Disagree; Strongly Disagree
  If you agree with the above statement, please give an example from your experience
9. I am aware of sustainability initiatives in my current healthcare workplace
  Strongly Agree; Agree; Neutral; Disagree; Strongly Disagree
  If you agree with the above statement, please give an example of a current workplace sustainability initiative you are aware of
10. I have come across sustainability initiatives in previous healthcare workplaces
  Strongly Agree; Agree; Neutral; Disagree; Strongly Disagree
  If you agree with the above statement, please give an example of a previous workplace sustainability initiative you are aware of
11. I have experience of being involved in sustainability initiatives in my current and/or previous workplaces
  Strongly Agree; Agree; Neutral; Disagree; Strongly Disagree
  If you agree with the above statement, please provide brief details of your experience in supporting a sustainability initiative
12. Based on my experiences of sustainable healthcare delivery so far, more input from workplace programs would be beneficial
  Strongly Agree; Agree; Neutral; Disagree; Strongly Disagree
  Please provide brief details of why you have agreed/disagreed
13. Based on my experiences of sustainable healthcare delivery so far, more input in education programs would be beneficial
  Strongly Agree; Agree; Neutral; Disagree; Strongly Disagree
  Please provide brief details of why you have agreed/disagreed
14. What one area of sustainability would you like to learn more about?
